# Existing and preferred organizational culture in Algerian power plants: a second look

**DOI:** 10.3389/fsoc.2025.1498129

**Published:** 2026-04-17

**Authors:** Raouf Kaouache, Mohammed Himrane, Gene A. Brewer, Abderrahmane Kaouache

**Affiliations:** 1 College of Arts, Humanities and Social Sciences, University of Al Dhaid, Sharjah, United Arab Emirates; 2 College of Humanities and Social Sciences, University of Mohammed Sedik Benyahia, Jijel, Algeria; 3 Faculty of Economics, Commercial and Management Sciences, University Mohammed Seddik Ben Yahia University, Jijel, Algeria; 4 Department of Public Administration and Policy, School of Public and International Affairs, University of Georgia, Athens, GA, United States; 5 Faculty of Humanities, Social and Islamic Sciences, Ahmed Draya University, Adrar, Algeria

**Keywords:** culture, existing organizational culture, preferred organizational culture, power plants, Algeria

## Abstract

**Introduction:**

Understanding organizational culture has become crucial for effective human resource management in organizations, and essential for developing more successful change strategies. Building on an earlier research project, this study aims to compare the existing and preferred organizational culture types from Harrison and Stokes perspective to measure the impact of power, role, and support cultures on managers’ aspirations to work within an achievement culture in four Algerian power plants.

**Methods:**

The study collects survey responses from 135 managers working in these power plants. This is a quantitative study which collects responses from 135 managers working in different organizational levels in these power plants. Data are analysed using the statistical package of social sciences, version 23 by comparing means and employing t tests and linear regression analysis.

**Results:**

The findings reveal significant differences between the existing and preferred culture types of power, achievement, and support cultures. Existing power culture scored higher than expected, while current achievement and support cultures scored lower than desired. In contrast, no significant differences between the current and preferred support cultures were found. In addition, the study revealed that preferred achievement culture is positively associated with existing power and support cultures.

**Discussion:**

In other words, managers working in traditional Arab cultures generally express stronger preferences for a culture that is more achievement oriented. The implications of these findings for Arab public management are discussed.

## Introduction

Recent literature in the field of organization and management studies sheds light on the importance of culture in organizations. Peters and Waterman (1984) revealed that strong culture is necessary for organizations’ excellence and efficiency. Several research shows that organizational culture is positively associated with organization performance (Martinez et al., 2015; Denison and Mishra, 1995; Byles et al., 1991). Also, other studies report positive relationships between culture and a handful of desirable outcomes, including sales growth, organizational efficiency, and employee satisfaction (Berson et al., 2008); commitment, satisfaction, and work-group cohesion (Odom et al., 1990) and employee turnover (Dwivedi et al., 2013; Biswas, 2009; Sheridan, 1992). Scholars and consultants view organizational culture as a stabilizer, a conservative force, and a means of making organizations more meaningful and predictable (Freiling and Fichtner, 2010). Organizational culture also helps researchers and managers understand planned changes in organizations (Ouchi and Wilkins, 1985). This means that managers use culture to convince employees to accept organizational changes. In addition, recent research realized that organizational culture impacts creativity and innovation within organizations (Ali et al., 2016). Finally, scholars point out that a perpetual learning process and flexible culture should help organizations to adapt and become more effective in complex, fast-paced, and culturally diverse environments (Schein, 2010, Martins and Martins, 2002; Jassawalla and Sashittal, 2002).

Organizational culture has been defined from different perspectives (Ouchi and Wilkins, 1985). Hofstede et al. (2005) looked to culture as the collective programming of the mind that distinguishes members of one group or category of people from others. Hofstede’s research focuses on society culture; however, it has direct implications for organizational culture. Thus, organizational culture is a tangible concept that makes a group of employees different from other groups. Also, organizational culture is defined as (1) pattern of basic assumptions, (2) invented, discovered, or developed by a given group, (3) as it learns to cope with its problems of external adaptation and internal integration, (4) that has worked well enough to be considered valid and, therefore (5) is to be taught to new members as the (6) correct way to perceive, think, and feel in relation to those problems. This definition provides insights into the life cycle of organizational culture, including how it forms, persists, and changes. Harrison and Stokes (1992) define culture as a pattern of beliefs, values, rituals, myths, and sentiments shared by the members of an organization. Culture is a shared tangible and intangible component between members and groups of an organization. It gives the organization a set of specifics that distinguish it from other organizations in terms of using power, rewards, motivation, treating others, and responding to the environment and values that organization members live by. Organizational culture is also categorized into explicit features, such as artifacts which could include uniforms and membership badges, and implicit features, such as values and assumptions shared by members of the culture. It is easy to observe, feel and note artifacts that are located at a high level of culture, and appear in the organization’s structure and processes. Organizational values are located at the middle level of culture and explain why the organization acts in a certain way. We can decipher values from the organization’s strategies, goals, and philosophies. The underlying assumptions, which started as values, exist at the deeper level of culture. It consists of perceptions and beliefs about human nature, the nature of relationships and activity, the nature of time, reality, and truth, ambiguity; and the organization’s relationship to its environment (Schein, 2010). Organizational culture is a multidimensional construction (Mesfin et al., 2020). In this regard, diagnosing the culture of organizations refers to a combination of multiple dimensions which can lead to different typologies. Several models have been developed by scholars from different perspectives to examine culture within organizations (Deal and Kennedy, 1982; Handy, 1993; Trompenaars and Hampden-Turner, 1997; Cameron and Quinn, 1999; Hofstede, 2004).

Scholars are placing increased interest in employee perceptions about organizational culture, particularly on the gap between existing and preferred culture. O'Reilly III et al. (1991) reported that congruence in individual and corporate values is positively associated with employee job satisfaction and organizational commitment. Also, Posner et al. (1985) found that employee consensus on prized organizational values makes a significant difference in employee well-being and organizational performance. In addition, Goodman and Svyantek (1999) showed that employee perceptions of discrepancies between actual and ideal culture are predictors of employee task and contextual performance. However, differences between what higher-ups perceive and the reality that employees experience leads to negative consequences. The 2016 Corporate Culture Chasm survey found that managers hold a much more positive view of their corporate culture than do employees. Along the same line, Macintosh and Doherty (2005) reported that a culture gap between leader intentions and employee perceptions is associated with increased employee turnover rates.

Currently, power plants management knows that organizational culture can help them to deal with deep and rapid changes that they are facing. For instance, American power plants tried to strengthen its culture by adopting a stakeholder engagement inward approach which helps to connect employees with management decisions and provide them work direction (Nessing, 2016). Also, the Chinese state has achieved the goal of restructuring and liberalizing the electricity industry, however, it failed to establish a functional regulatory system because of its bureaucratic inflexibility, limited competition, and monopoly control. Algerian power plants have implemented a lot of measures to develop its cultures in terms of leadership and management styles, as well as type of technology to deal with changes in employee’s attitudes, values, and expectations, and to meet the growing demand for electricity consequently. This study aims to identify the cultural gap between existing and preferred organizational culture according to Harrison and Stokes model, and identify the impact of existing power, role, and support organizational cultures on preferred achievement organizational culture.

## Theory on existing and preferred organizational culture

Several studies have been conducted to identify existing organizational culture within different contexts. Hofstede (1983) realized that Arab organizations are characterized by large power distance, and centralization is rooted in the Arab leader’s mental programming which permits them to lead based on autocracy. This means that leaders in Arab organizations believe that centralizing power at the top management level help to control work and improve organizational performance. Other researchers have confirmed that Arab public organizations are characterized by centralized decision-making structures (Sharda and Miller, 2001; Miller and Sharda, 1997; Mansfield and Zeffane, 1983; Badran and Hinings, 1981; Ayoubi, 1975). Therefore, many Arab organizations adopt hierarchical structures which give top executives the right to make important organizational decisions. Al-Yahya (2009) suggests that managers in Arab public organizations resist formal power-influence sharing practices and try to retain authority and decision making because they believe that power distribution and equalization among members may reduce the influence of managers. This pattern has been supported by other research that found a general tendency among Arab executives to be detail-oriented and to rely on personal power to manage their organizations (Ali, 1989; Al-Rasheed, 2001; Badawy, 1980).

On the other hand, research findings from Arab countries reveal persistent differences in preferred and existing organizational cultures. Branine and Pollard (2010) shed light on the gap between non-Islamic traditional management derived from Arab national culture, and the values and norms of western management practices that managers often adopt in Arab organizations. Also, Sabri (2004) noted that employees in Jordan prefer working in organizations that are more oriented toward an achievement culture. Other research findings reported that human resources officials emphasize the importance of high task—high employee management on organizational effectiveness in Arab countries (Badawy, 1980). In the same line of these research results, Ali (1989) found that managers in Kuwait are highly aware about the effectiveness of participative decision-making style in public organizations. Furthermore, according to other research, the consultative and pseudo- consultative are the most preferred styles in Arab Gulf managerial orientations (Al-Yahya, 2009; Ali, 1989; Al-Jafary and Hollingsworth, 1983). Recent research found that majority of managers in Saudi Arabian public organizations prefer working according to participative rather than consultative management practices, and that a trend toward developing a more participatory style of management is evolving. Other research findings also reported that employers prefer more participation and consultation in business functioning, as decentralized management enables them to reach bottom-line efficiency and high employee motivation (Casari and Plott, 2003).

For Algerian organizations, Zeffane (1988) research revealed that central planning impedes the adoption of participative management reforms. Also, he found that top managers tend to dominate decision-making processes (Zeffane, 1989). Other research showed that most middle- and lower-level managers hold functional and consultative authority, however, they are not empowered to make important decisions (Ancer, 2008). Ancer (2008) also corroborated employee aspirations for a higher level of participation in Algerian organizations, which mean that managers and employees want more participation. According to Mercure et al. (1998) leaders and subordinates in Algerian organizations prefer high level of formalization. This means that managers and employees agree on the type of culture with – low centralization and high formalization. Mercure et al. (1998) also noted a strong cultural gap between management orientation inspired from the Soviet model and employee’s aspiration which value the cultural fund of Algerian society. They also pointed out that managers in Algeria tend to retain authority, which is not compatible with employee aspirations for more participation. Along the same line, Kaouache et al. (2020) demonstrated that major differences between the existing and the preferred culture in Algerian organizations. Consequently, this cultural gap could create different challenges for management and employees, and recent research has noted the importance of contextualizing management practices to achieve efficiency in human resources management (Brewster et al., 2015; Budhwar et al., 2016; Chourasia et al., 2024; Baojing et al., 2025; Soncco et al., 2025; Cyfert et al., 2025).

**Figure 1:**
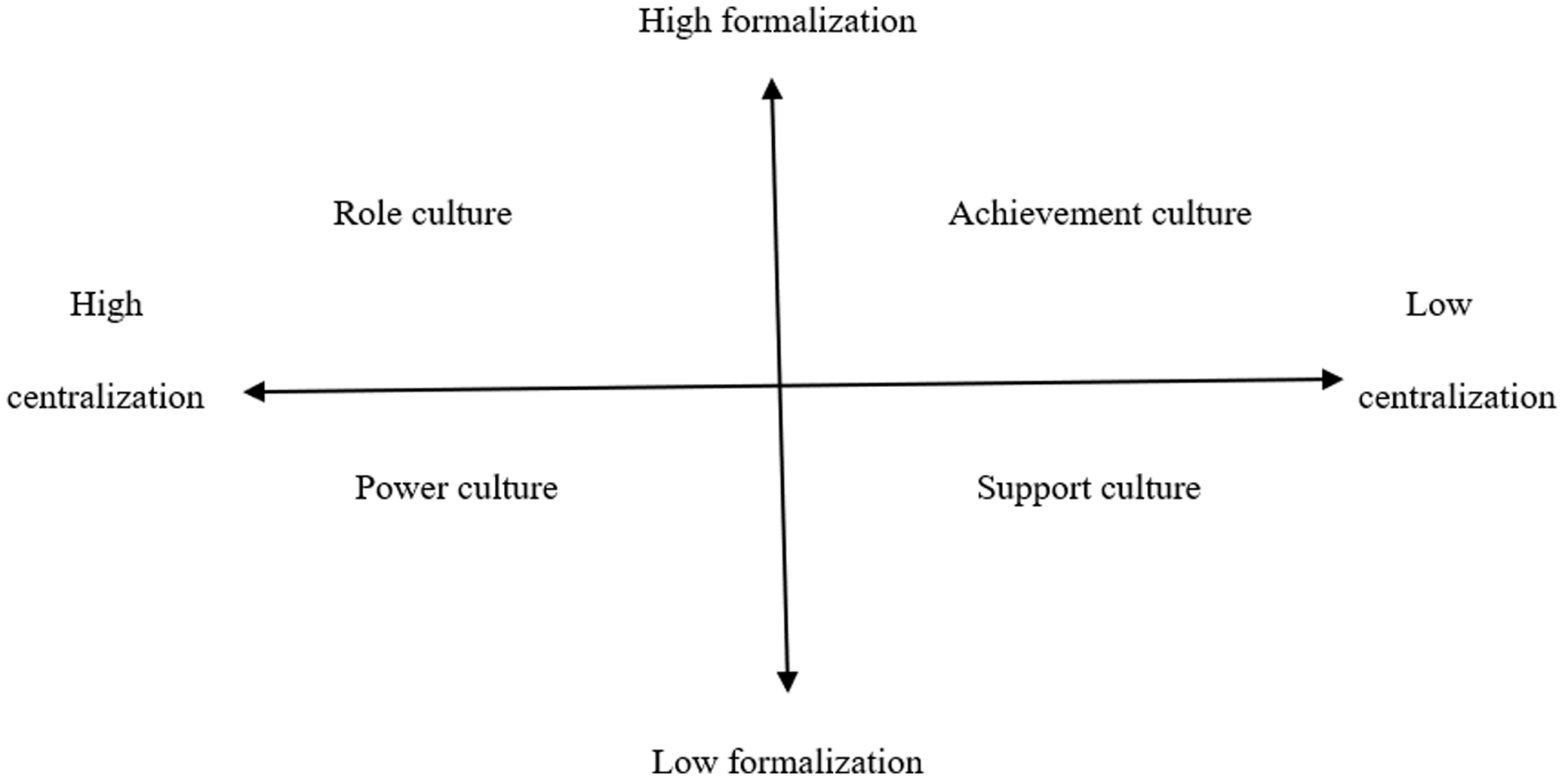
Types of organizational culture.

Several studies have examined the topic of existing and preferred organizational culture from different perspectives. Harrison and Stokes (1992) developed an instrument based on centralization and formalization dimensions in organizations. Harrison and Stokes (1992) noted that traditional societies legitimize hierarchy much more than industrialized democracies, which means that people accept inequality of access to resources. They created four types of culture within organizations named: power culture, role culture, achievement culture, and support culture with different characteristics. The four types of culture are shown in Figure 1.

Power culture is a combination of high centralization and low formalization. In this type of culture, power is wielded by a few leaders who control work and influence employees through their personal abilities rather than rules and procedures. Thus, managers hold the right to dominate important decisions and keep the right to interfere in different work situations (Harrison, 1993). For power culture, the first hypothesis is formulated as follows.

*H1a*: There is significant difference between existing and preferred power culture in Algerian power plants from the manager’s perspective.

Role culture is the result of high levels of centralization and formalization, wherein leaders rely on rules and procedures to manage work. Organizations with this type of culture have a hierarchical structure that concentrates power in top managers, who delegate authority to subordinates to influence and maintain control over the organization (Harrison and Stokes, 1992). In this type of culture, job descriptions are clearly defined, and tasks are strictly prescribed and regulated by a set of rules, processes, and procedures that should be respected when making bureaucratic decisions. For role culture, the second hypothesis is formulated as follows.

*H1b*: There is significant difference between existing and preferred role culture in Algerian electric power plants from the manager’s perspective.

Achievement culture is a combination of low centralization and high formalization, where managers prioritize empowering subordinates, teamwork, and mutual control. Management typically seeks to empower employees and promote collaborative efforts toward shared objectives, guided by clearly defined rules and procedures. Within this organizational framework, employees are regarded as experts in decision-making, active participants in teamwork, and contributors to mutual oversight and accountability. For role culture, the third hypothesis is formulated as follows.

*H1c*: There is significant difference between existing and preferred achievement culture in Algerian power plants from the manager’s perspective.

Support culture exhibits low levels of both centralization and formalization, wherein managers rely on mutual trust to manage work (Harrison and Stokes, 1992). Mutual trust between top management and employees enables the latter to exercise a high degree of decision-making authority, autonomy in organizing and managing their tasks, and the ability to influence colleagues in developing competencies and reaching consensual decisions. For role culture, the fourth hypothesis is formulated as follows.

*H1d*: There is significant difference between existing and preferred support culture in Algerian power plants from the manager’s perspective.

These hypotheses address comparisons between existing and preferred organizational cultures according to Harrison and Stokes model which contains four types of organizational culture named power, role, achievement, and support.

**Figure 2:**
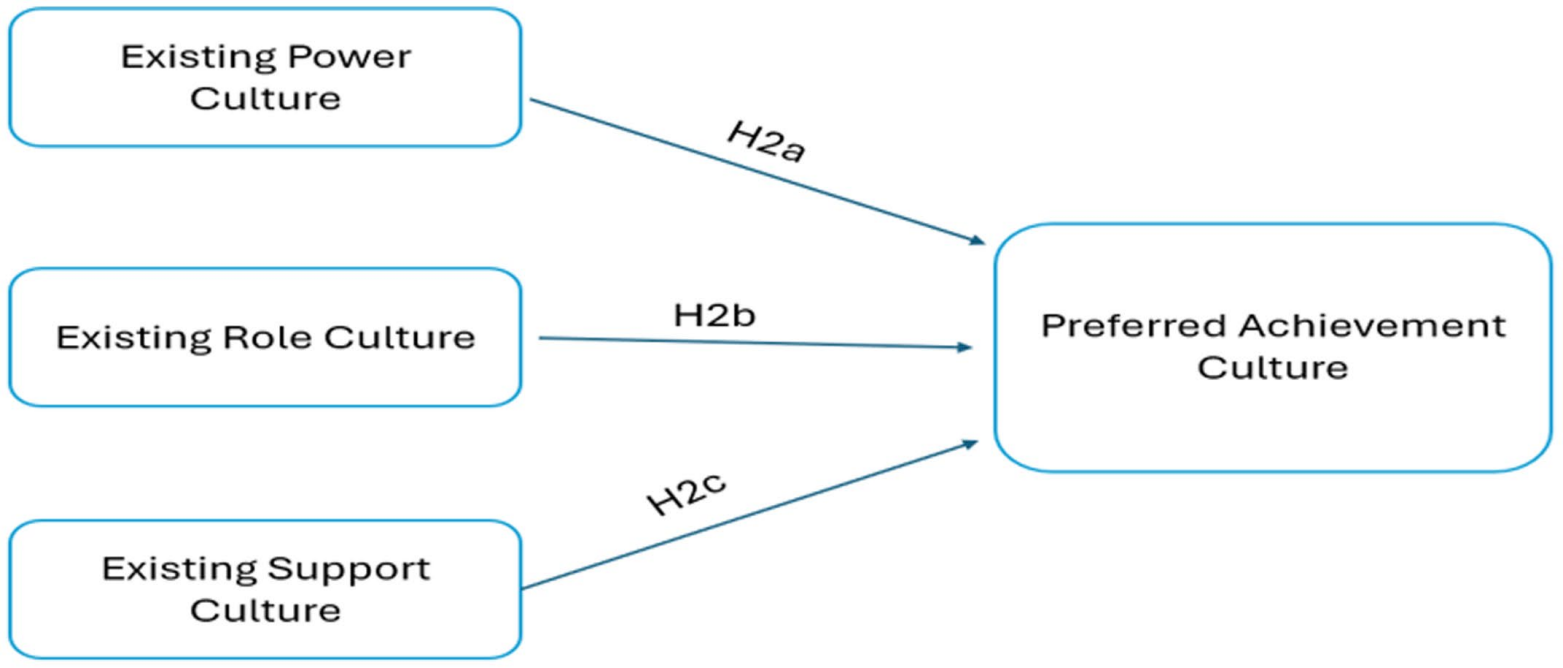
The impact of existing power, role, and support culture on preferred achievement culture.

Organization culture is a mixture of the four existing levels of centralization and formalization occurring at different levels. This means that various forms of power, role, achievement, and support cultures coexist within organizations which have important implications for measuring cultures and bridging the gap between the existing and preferred organizational culture. The research model adopted for this study allows managers to identify the existing organizational culture rates which differ from the preferred organizational culture, and measure the impact of existing power, role and support organizational cultures on preferred achievement organizational culture (Figure 2).

Alignment of existing and preferred cultures is important for both organizational cultures. Clearly, managers should understand their relationship alike. Harrison and Stokes (1992) typology permits researchers to measure cultural gaps and understand why they exist by mapping the dynamic relationship between centralization and formalization in organizations.

These hypotheses assess the association between existing power, role, and support organizational cultures on preferred achievement organizational culture of managers in Algerian power plants.

*H2a*: Power culture is positively associated with preferred achievement culture in Algerian power plants from the manager’s perspective.

*H2b*: Existing role culture is positively associated with preferred achievement culture in Algerian power plants from the manager’s perspective.

*H2c*: Existing support culture is positively associated with preferred achievement culture in Algerian power plants from the managers’ perspective.

## Research methodology

### Sample selection and survey administration

The survey was administered with 135 managers working in four power plants in Algeria. Before data collection, researchers got the ethical certificate from the research ethics committee of Abdelhamid Mehri University, Constantine, Algeria. Verbally informed consent was obtained because we conducted a self-administered questionnaire. Participants were informed about the consent process on the first page of the questionnaire, and they agreed voluntarily to participate in the research by filling in the survey. This filling is considered as an acceptance for participation. Alongside, confidentiality and privacy of participants’ data were ensured. Data that support the findings of this study are available from the corresponding author K R and you can ask for based on reasonable request.

A purposive sampling method was used to select four Algerian power plants (Jijel, Bejaia, Skikda, and Algiers), based on organizational willingness to authorize participation. Within these sites, a stratified purposive sampling technique was applied to ensure diverse representation across managerial demographic characteristics (e.g., gender, age, education, seniority, experience, and salary). This sampling approach is appropriate for exploratory organizational culture research where representativeness across key professional subgroups is critical, and full randomization is limited due to access and organizational constraints. Prior to full deployment, the questionnaire was pilot tested with 25 managers from different hierarchical levels to assess its validity and clarity. Subsequently, 251 questionnaires were manually distributed in May 2016. A total of 144 responses were received; however, nine were excluded due to incompleteness. This resulted in a final dataset of 135 valid questionnaires, corresponding to a response rate of 53.78%.

### Survey measures and items

This study adopted Harrison and Stokes organizational culture instrument (1993) for measuring organizational culture, which is well-established in organizational behavior research and provides a multidimensional evaluation of organizational culture types through 15 subscales measuring power, role, achievement, and support cultures. To ensure content validity, it was pilot tested with 25 managers representing different hierarchical levels in Algerian power plants, and feedback from this phase was used to refine linguistic and contextual relevance. Consequently, the survey achieved strong face validity for the Algerian industrial context. Also, the questionnaire is reliable and valid according to the Spearman-Brown formula (Harrison, 1993). For the existing culture, managers are asked to rank the alternatives. A score of “4” means the items come closest to describing the way things are in their production units, and “3,” “2,” and “1” indicate less close fits in descending order. Managers are also asked to rank the preferred culture alternatives by scoring “4” as their most preferred option, and “3,” “2,” and “1” signifying the option they prefer least in descending order. Data is entered using the statistical package of social sciences (SPSS) version 23. Internal consistency reliability of the questionnaire was verified using Cronbach’s alpha. All dimensions exceeded the 0.60 threshold commonly recommended for exploratory research, and two exceeded the 0.70 threshold suitable for confirmatory studies. Power (existing: *α* = 0.786; preferred: α = 0.840), Role (existing: α = 0.701; preferred: α = 0.711), Achievement (existing: α = 0.655; preferred: α = 0.711), Support (existing: α = 0.670; preferred: α = 0.715). Also, reliability of data has been tested using paired samples *t**-***test to examine differences between existing and preferred scores, multiple regression analysis to predict the impact of existing cultures on preferred achievement culture, and variation inflation factor to detect multicollinearity which revealed that all were below the conservative cut of 0.5.

## Findings

The sample of 135 respondents contains 89.60% male. Managers’ ages ranged from 21 to over 55, with 41.80% between 35 and 45 years old. A large percentage of the managers were married (81.30%) and university graduates (91.80%). Their work experience was: 10–15 years, 40.30%; over 30 years, 30.60%; 15–20 years and 20–25 years, 11.90%; and 25–30 years and less than 5 years with 4.5 and 0.7%, respectively. More than half of the managers earn a monthly salary that ranges between 450 and 500 USD. Table 1 presents these personal and professional characteristics of the sample, which are typical of managers in Algerian power plants. Table 2 reports the means, standard deviations, and results of T tests on differences in existing and preferred organizational culture.

**Table 1:** Descriptive statistics of personal and occupational characteristics.

Personal and occupational characteristics	Variables	Frequency	Percent
Gender	Male	120	89.6
	Female	14	10.4
Age	Less than 35 years old	40	29,9
	From 35 to less than 40 years old	23	17,2
	From 40 to less than 45 years old	33	24,6
	From 45 to less than 50 years old	19	14,2
	From 50 to less than 55 years old	12	9,0
	55 years and over	7	5,2
Familial status	Married	109	81.3
	Single	25	18.7
Certificate	Middle school certificate	4	3,0
	Secondary education certificate	7	5.2
	Bachelor’s degree	30	22.4
	State engineer certificate	74	55.2
	Master’s degree	4	3.0
	Specialized Postgraduate Certificate	7	5.2
	Ph. D	1	0.7
	Senior technician certificate	7	5.2
Seniority	Less than 5 years	1	0.7
	From 10 to less than 15 years old	54	40.3
	From 15 to less than 20 years old	16	11.9
	From 20 to less than 25 years old	16	11.9
	From 25 to less than 30 years old	6	4.5
	30 years and over	41	30.6
Pay	From 40,000 to 44,999 DZD	5	3.7
	From 45,000 to 49,999 DZD	21	15.7
	From 45,000 to 49,999 DZD	13	9.7
	From 50,000 to 54,999 DZD	16	11.9
	From 55,000 to 59,999 DZD	9	6.7
	From 60,000 to 64,999 DZD	29	21.6
	65,000 DZD and more	41	30.6
Total		134	100

**Table 2:** Differences in existing and preferred organizational culture.

Types of culture	Category	Mean	SD	*T*-test (2-tailed)	Sig
Power culture	Existing	2.94	0.332	12.239	0.000
	Preferred	1.89	0.314		
Role culture	Existing	2.78	0.334	1.192	0.235
	Preferred	2.73	0.336		
Achievement culture	Existing	2.27	0.463	−12.284	0.000
	Preferred	2.98	0.593		
Support culture	Existing	1.99	0.593	−6.625	0.000
	Preferred	2.37	0.485		

Managers across the four power plants surveyed in this study provided the following mean ratings for existing organizational culture types: *power culture* (*M* = 2.94), *role culture* (*M* = 2.78), *achievement culture* (*M* = 2.27), and *support culture* (*M* = 1.99). In contrast, their preferred organizational cultures reflected a notable shift in emphasis, with a lower mean rating for *power culture* (*M* = 1.89) and higher preferences for *role culture* (*M* = 2.73), *achievement culture* (*M* = 2.98), and *support culture* (*M* = 2.37). As presented in Table 2, the dispersion of responses was relatively low for both existing and preferred culture types, indicating a consistent pattern in managerial perceptions. The standard deviation for existing culture types ranged from 0.332 to 0.593, suggesting limited variability and convergence in perceptions of the current organizational culture. Similarly, the standard deviation for preferred culture types ranged from 0.314 to 0.593, implying a comparable level of agreement among managers regarding the cultural attributes they aspire to see adopted within their organizations.

The results of the *t*-test analysis, as presented in Table 2, indicate statistically significant differences between the existing and preferred organizational culture types in three out of the four examined categories. Specifically, a significant difference was observed between the existing and preferred power culture, with managers identifying it as the most dominant current culture but expressing a clear preference for it to be the least dominant. These findings provide empirical support for Hypothesis 1a. In contrast, no statistically significant difference was found between the existing and preferred role culture, indicating that managerial perceptions regarding this culture type are stable. Consequently, Hypothesis 1b is not supported. The analysis further reveals a significant discrepancy between the existing and preferred achievement culture, suggesting that managers are dissatisfied with its current level and would prefer it to become the most dominant culture type within the organization. These findings validate Hypothesis 3. Lastly, the support culture was identified as the least dominant in the current organizational context; however, managers expressed a preference for its increased prominence. This difference supports the acceptance of Hypothesis 4.

The variation inflation factor among the four existing organizational culture types reveals no severe multicollinearity. This means that ordinary least squares multiple regression analysis is appropriate for analyzing the data and testing the study hypotheses. The results of multiple regression analyses test presented in Table 3 reveal a significant impact of two existing organizational culture types on preferred achievement culture with an adjusted R^2^ value of 0.192 which is statistically significant at the 0.01 level. This means that 19.2% of the variation in preferred achievement culture is explained by existing power culture and role cultures.

**Table 3:** Results of multiple regression analyses predicting preferred achievement culture.

Dependent variable	Independent variable	Regression coefficient (β)	Standard error	*T*
Existing power culture	Preferred achievement culture	0.339^***^	0.090	3.750
Existing role culture		0.458***	0.172	2.665
Existing support culture		0.156	0.161	0.968
	*R* ^2^	0.247^**^		
	Adjusted *R*^2^	0.192		
	*F*	4.519^***^		

*N* = 135; *p < 0.05, **p < 0.01, ***p < 0.001.

Among the existing organizational culture types, power culture is positively associated with preferred achievement culture. That is, Managers exhibit high level of centralization and respect of formal procedures reported higher level of achievement culture than those who did not. As a result, hypothesis 2a is supported. Also, the results indicate that managers who perceived high level of role culture were more likely to prefer achievement culture within power plants in Algeria, supporting hypothesis 2b. Conversely, no statistically significant correlation was found between existing support culture and preferred achievement culture, meaning hypothesis 2c is rejected.

## Implications

The results of this study reveal that managers’ perceptions about the existing and preferred organizational culture types are different. While power is the most spread type of organizational culture in the Algerian power plants, most managers consider this culture type the least preferred. This suggests that leaders in these organizations feel constrained by being unempowered and need to get higher management permission for managing different work situations. Ledimo (2013) found that power culture is the most dominant type in manufacturing organizations in South Africa. Also, power plants work in relatively high role culture environments, according to managers. This result reflects the importance of job descriptions and formal procedures to manage work in Algerian power plants. Harrison and Stokes (1992) conducted a comparative study of organizational culture and realized the importance of positions as a source of power for managers. Employees do not necessarily benefit from this aspect of role culture. In addition, the results of this study show that more managers perceive existing achievement culture as relatively low; however, they prefer to work in power plants that prioritize this type of culture. This means that managers seek to be more empowered for decision-making and have more autonomy at work. This result is consistent with Sabri (2004) findings which revealed that employees in Arab firms prefer to work in achievement-oriented organizations that place importance on formal goals and employee performance. Finally, the results reveal that support culture is perceived as the least dominant existing culture, and more managers would prefer it to be more dominant. Other studies show that power plant employees hold similar views (Kaouache et al., 2020). It should be noted that managers believe adherence to group norms, excessive focus on procedures, and the suppression of conflict impede their ability to take appropriate and effective initiatives in the workplace.

The findings indicate that existing power and role cultures exert a significant influence on the preferred achievement-oriented culture within public power plant organizations in Algeria. Specifically, power and role cultures were identified as significant predictors of managers’ aspirations to operate within an achievement-oriented organizational culture—one characterized by a high degree of decentralization and formalization. Among these, power culture emerged as the most influential predictor. These insights offer practical implications for public power plants aiming to enhance achievement-oriented practices: organizational strategies should prioritize the development of achievement cultures while strategically balancing centralization and formalization across hierarchical levels. Additionally, although managers reported a moderate presence of support culture within their organizations, this cultural dimension did not exhibit a statistically significant relationship with the preferred achievement culture. These results suggest that managers favor organizational structures that promote both decentralization and formalized processes.

The results imply other organizational strategies that should be adopted by top management to enhance the adoption of achievement culture. Examples include encouraging power delegation and empowering middle- and low-level managers to handle work requirements and adopt a performance-based system which incentivizes excellence at work. Furthermore, management should develop a strategic vision of human resources and prioritize creating and sustaining an environment that is receptive to change.

## Conclusion

This study aimed to identify differences between the existing and preferred organizational culture according to the Organizational Culture Questionnaire (OCQ) developed by Harrison and Stokes (1992), and to measure the impact of existing power, role, and support cultures on preferred achievement culture in Algerian power plants. The results revealed differences between the existing and preferred power, achievement, and support cultures from the managers perspective. In contrast, there was no significant difference between existing and preferred role culture. Managers at different levels felt that power culture is too dominant. This means that power is seen as too centralized, which is not compatible with the managers’ preferences. Also, the existing achievement culture scored less than what power plant managers prefer. Managers prefer to get more priority to achieving their formal missions and goals, and by selecting the right human resources, then motivating and developing workers in line with the organization’s strategy. In addition, managers want to raise the level of mutual trust between employees and managers to achieve a higher level of support culture within Algerian power plant organizations. On the other hand, existing and preferred organizational role culture seems congruent, which means that work control based on rules and formal are aligned with managers’ expectations.

To achieve a high level of achievement culture in Algerian power plants, our findings revealed that existing power and role organizational cultures are significant predictors. The high centralization inherent in power and role organizational cultures may urge managers to prefer decentralization, which would increase their chance to participate in decision making. In addition, the high and medium level of formalization associated with power and role organizational cultures may underlie the high preference for formalization among managers in Algerian power plants. Management should respond to the managers’ expectations in terms of increasing participation and respect for rules and procedures.

To transform Algerian power plants into more achievement-oriented organizations, management should adopt a strategy that prioritizes task performance by establishing clear goals, determining roles, and encouraging creativity among managers. These priorities are especially important because of unpredictable rising demand for electricity, especially in the summer season, and the likely changes in aspirations, culture, potential and behaviors that Algerian power plants will likely experience as the market grows more dynamic. This study can be a starting point for future research projects on organizational culture involving more power plants in the west and the south of Algeria, and for examining other elements of culture within power plants. Future research may also shed light on the mediating role of sociodemographic variables regarding the relationship between existing and preferred organizational cultures, and the impact of existing cultures on maintaining preferred role culture in Algerian power plants.

This study has several limitations in terms of theory and methodology. First, future research could broaden the research model to include other aspects of organizational culture. Second, the study used a questionnaire to collect data about perceptions regarding organizational culture, while other research tools as interviews are important to get qualitative data about organizational culture. Third, the study has been conducted in four power plants located in the northeast and center of Algeria, and broadening research field to other regions could provide additional insights about differences on existing and preferred organizational cultures.

## Data Availability

The original contributions presented in the study are included in the article/supplementary material, further inquiries can be directed to the corresponding author.
